# Developmental expression of catecholamine system in the human placenta and rat fetoplacental unit

**DOI:** 10.1038/s41598-024-57481-5

**Published:** 2024-03-23

**Authors:** Rona Karahoda, Veronika Vachalova, Ramon Portillo, Filip Mahrla, Mireia Viñas-Noguera, Cilia Abad, Frantisek Staud

**Affiliations:** Department of Pharmacology and Toxicology, Faculty of Pharmacy in Hradec Králové, Charles University, Prague, Czech Republic

**Keywords:** Placenta, Monoamines, Trophoblast, Metabolism, In vitro models, Hormones, Biochemistry, Endocrinology

## Abstract

Catecholamines norepinephrine and dopamine have been implicated in numerous physiological processes within the central nervous system. Emerging evidence has highlighted the importance of tightly regulated monoamine levels for placental functions and fetal development. However, the complexities of synthesis, release, and regulation of catecholamines in the fetoplacental unit have not been fully unraveled. In this study, we investigated the expression of enzymes and transporters involved in synthesis, degradation, and transport of norepinephrine and dopamine in the human placenta and rat fetoplacental unit. Quantitative PCR and Western blot analyses were performed in early-to-late gestation in humans (first trimester vs. term placenta) and mid-to-late gestation in rats (placenta and fetal brain, intestines, liver, lungs, and heart). In addition, we analyzed the gene expression patterns in isolated primary trophoblast cells from the human placenta and placenta-derived cell lines (HRP-1, BeWo, JEG-3). In both human and rat placentas, the study identifies the presence of only PNMT, COMT, and NET at the mRNA and protein levels, with the expression of PNMT and NET showing gestational age dependency. On the other hand, rat fetal tissues consistently express the catecholamine pathway genes, revealing distinct developmental expression patterns. Lastly, we report significant transcriptional profile variations in different placental cell models, emphasizing the importance of careful model selection for catecholamine metabolism/transport studies. Collectively, integrating findings from humans and rats enhances our understanding of the dynamic regulatory mechanisms that underlie catecholamine dynamics during pregnancy. We identified similar patterns in both species across gestation, suggesting conserved molecular mechanisms and potentially shedding light on shared biological processes influencing placental development.

## Introduction

Catecholamines dopamine, norepinephrine, and epinephrine are physiologically active molecules that act as neurotransmitters and hormones implicated in numerous physiological processes. During the prenatal period, they are among the first neurochemicals to develop in the embryo with functions essential for fetal development^1^. Importantly, growing evidence suggests a link between in utero neurotransmitter homeostasis and developmental programming of the fetal heart, brain, kidneys^2–4^; nonetheless, mechanistic causes are still poorly understood.

During pregnancy, fetal organs are dependent on the placenta as a conduit for exchange and a mediator responsive to in utero stressors. A tight communication exists between the placenta and fetal organs often referred to as "placenta-brain", "placenta-heart", or "placenta-gut" axis^4–6^. Although the placenta represents a non-neuronal tissue, its unique neuroendocrine milieu has recently gained considerable attention due to the expression of proteins involved in the synthesis, degradation, and transport of serotonin^7,8^. During early brain development, the placenta is the sole source of serotonin^8^, whereas at term, it provides a protective mechanism against high serotonin concentrations in the fetoplacental unit^9^. Nonetheless, the effect of the placental biochemical environment on the catecholaminergic system development is scarcely understood.

**Figure 1:**
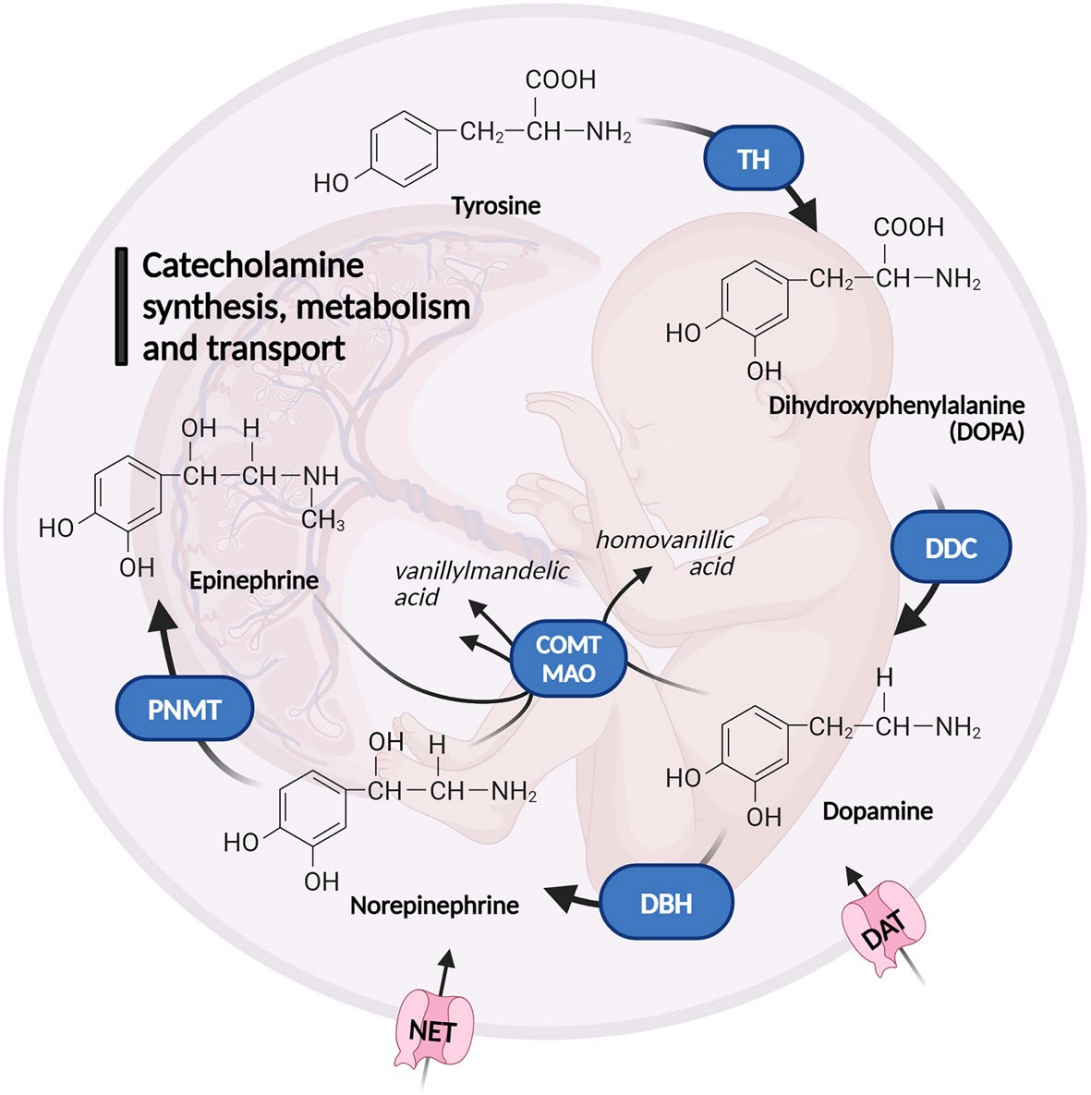
Schematic depiction of key enzymes and transporters involved in catecholamine synthesis, metabolism, and transport in the fetoplacental unit. Created in BioRender.com. *COMT* catechol-o-methyltransferase, *DAT* dopamine transporter, *DBH* dopamine β-hydroxylase, *DDC* dopa decarboxylase, *MAO* monoamine oxidase, *NET* norepinephrine transporter, *PNMT* phenylethanolamine N-methyltransferase, *TH* tyrosine hydroxylase.

The cellular catecholamine handling can be divided into synthesis, degradation, and transport (Fig. 1). Catecholamine synthesis begins with the hydroxylation of the amino acid L-tyrosine to yield L-dihydroxyphenylalanine (L-DOPA), a step mediated by tyrosine hydroxylase (TH). Subsequently, L-DOPA is converted to dopamine by the action of the dopa decarboxylase (DDC). Further hydroxylation of dopamine by the enzyme dopamine β-hydroxylase (DBH) yields norepinephrine, which can be subsequently methylated by phenylethanolamine N-methyltransferase (PNMT) to generate epinephrine. Catecholamine degradation occurs either by monoamine oxidase (MAO) or catechol-o-methyltransferase (COMT), which are highly expressed in the placenta^9,10^. Ultimately, the transfer of dopamine and norepinephrine is mediated via the dopamine transporter (DAT) and norepinephrine transporter (NET), respectively. While the placenta functionally expresses NET^11^, placental DAT expression has not been reported to date.

In addition to its role in synthesizing serotonin, accumulating evidence suggests that the placenta may serve as a potential source of catecholamines and play a regulatory role in modulating their levels within the fetoplacental unit. Specifically, dopamine was shown to exert important paracrine functions within the placenta by inhibiting the production of human placental lactogen and human chorionic gonadotrophin (hCG)^12^. Additionally, the human placenta contains receptors belonging to the D_2_-like receptor family, which specifically bind dopamine^13^. Similarly, norepinephrine regulates hCG production in first-trimester trophoblast tissue^14^. Lastly, the placenta has been regarded as the dominant organ implicated with fetal catecholamine clearance^10^. Nonetheless, controversies remain on the placental expression of proteins involved in catecholamine handling^4,15^. Moreover, the catecholaminergic system may be subjected to physiological regulation during gestation, to adapt to placental and fetal needs. Current knowledge is limited in understanding potential transitions of developmental control between the placenta and embryo.

Animal models have been indispensable in exploring the prenatal environment through long-term administration of substances, in-depth placental function evaluations, and estimation of fetal exposure and toxicity^16–19^. However, the diversity in placental structure across mammalian species necessitates a careful consideration of various classification systems. In rodents and lower primates, the labyrinthine arrangement represents a sophisticated type of maternal–fetal interdigitation^20^, whereas humans exhibit a villous type of placentation with tree-like chorionic villi^21^. The hemochorial placental type, where the chorionic surface is in direct contact with maternal blood, is shared by both rodents and humans, with differences in the number of trophoblast layers^22^. Despite interspecies differences, the Wistar rat stands out as a suitable model for studying placental tryptophan and serotonin metabolism/transport^9,23–25^. Recognizing the indispensable role of animal research in reproductive biology, it is imperative to investigate and compare catecholamine handling in rats and humans. This study aims to provide valuable insights into the dynamic regulatory mechanisms governing catecholamine dynamics during pregnancy and enhance our understanding of shared biological processes influencing placental and fetal development.

## Methods

### Human placenta collection

Human placentas were collected at the University Hospital in Hradec Kralove, Czech Republic. First-trimester samples were obtained upon elective interruption of healthy pregnancies (gestational age at delivery 8–11 weeks) and are the same cohort as used in our previous study^7^. Term tissues were collected from uncomplicated healthy pregnancies after delivery (gestational age at delivery 38–40 weeks). The cohort comprised a total of 13 first-trimester and 28 term samples. All samples were utilized for gene expression studies, with a subset of n = 5 from each group additionally used for protein analysis. Patient characteristics are provided in Supplementary Table S1. All experiments were performed following the Declaration of Helsinki. Placental sample collection was obtained upon women's written informed consent and with the approval of the University Hospital Research Ethics Committee (201006 S15P).

### Rat placenta and fetal organ collection

The cohort of placental tissue and fetal organs (kidney, liver, brain, intestines, heart, and lung) used in this study, were collected at gestational days (GD) 15, 18, and 21 from Wistar rats, as described in a previous investigation^24^. Briefly, the positive copulatory plug of sperm after overnight mating was identified as gestational day 1. At specific pregnancy time-points, the rats were anesthetized with 40 mg/kg pentobarbital, tissues were dissected, and used for homogenate preparations or snap-frozen at − 80 °C. The placenta cohort included a total of 5 samples each for GD15, GD18, and GD21. All samples were employed for gene expression studies, and a subset of n = 4 from each group was additionally used for protein analysis. On the other hand, the fetal organ cohort consisted of at least 4 samples for each GD. All samples were derived from individual biological replicates, and the samples from each dam were pooled. All procedures were compliant with the ARRIVE guidelines and following the U.K. Animals (Scientific Procedures) Act, 1986 and associated guidelines of EU Directive 2010/63/EU for animal experiments. Experiments were approved by the Ethical Committee of the Faculty of Pharmacy in Hradec Kralove (MSMT-4312/2015-8).

### Cells

Primary trophoblast (PHT) cells were isolated from the human term placenta using three steps of enzymatic digestion and Percoll gradient separation, as previously described^7^. Cell purity was determined by staining with specific cell markers and flow cytometry analysis. Only cells with purity > 90% were used in the study. Cells were cultivated in DMEM (high glucose, GlutaMAX) supplemented with 10% FBS, penicillin 100 U/ml, and streptomycin 0.1 mg/ml. Cells were cultured for 12 h (cytotrophoblast, CTB stage) or 72 h (syncytiotrophoblast, STB stage), with a daily change of the medium.

The human choriocarcinoma cell lines BeWo and JEG-3 were obtained from the European Cell Culture Collection (United Kingdom). BeWo cells were cultured in Ham F-12 medium supplemented with 10% FBS, whereas JEG-3 cells were cultured in MEM supplemented with 10% FBS. To induce differentiation, the BeWo cell line was subjected to forskolin (20 µM) for 24 h. The rat trophoendodermal cell line HRP-1 was obtained as a generous gift from Dr. Michael Soares, University of Kansas Medical Center, USA. Cells were cultured in RPMI 1640 medium supplemented with 10% FBS, 1 mM sodium pyruvate, and 50 µM β-mercaptoethanol.

All cells were cultured at 37°C, 5% CO_2_, 95% N_2_, with daily change of the medium. The experiments were conducted with 4 biological replicates, each analyzed in technical triplicates. To assess the differentiation success of PHT and BeWo cells into the STB stage, the concentration of human chorionic gonadotropin (hCG) was monitored using an ELISA kit (Sigma Aldrich, USA). The results are presented in Supplementary Fig. S1.

### RNA isolation, reverse transcription, qPCR analysis

RNA was isolated from the whole tissue/cells using TRI Reagent (Molecular Research Center, USA). RNA concentration and purity were determined spectrophotometrically using NanoDrop 1000 (Thermo Fisher Scientific, USA). Reverse transcription was carried out using the iScript Advanced cDNA Synthesis Kit (Bio-Rad, United States), according to the manufacturer's instructions. Subsequently, PCR analysis was performed using predesigned TaqMan Assays (Thermo Fisher Scientific, USA). Amplification of 12.5 ng/µl cDNA was done in QuantStudio 6 (Thermo Fisher Scientific, USA), following the thermal cycling parameters for TaqMan Universal Master Mix II, no UNG (Thermo Fisher Scientific, USA). For human tissues/cells, the following genes were used: *TH* (Hs00165941_m1), *DDC* (Hs01105048_m1), *DBH* (Hs01089840_m1), *PNMT* (Hs01557113_g1), *COMT* (Hs00241349_m1), *NET/SLC6A2* (Hs00426573_m1), *DAT/SLC6A3* (Hs00997374_m1). On the other hand, for rat tissues/cells, the following genes were used: *Th* (Rn00562500_m1), *Ddc* (Rn01401189_m1), *Dbh* (Rn00565819_m1), *Pnmt* (Rn01495589_g1), *Comt* (Rn01404927_g1), *Net/Slc6a2* (Rn00580207_m1), Dat/Slc6a3 (Rn00562224_m1). Target gene expression was normalized against two reference genes using the 2^ΔΔCt^ method^26^. The sample with the lowest Ct value or the untreated control (in cell differentiation studies) was chosen as the calibrator. For human samples, *GAPDH* (Hs02786624_g1) and *B2M* (Hs00187842_m1) were used, whereas for rat samples *Gapdh* (Rn01775763_g1) and *Ywhaz* (Rn00755072_m1). For each qPCR sample we conducted technical triplicates.

### Protein extraction and western blot analysis

Collected tissues (as whole) were washed in saline (3 mL/g) and homogenized in a solution containing 250 mM sucrose, 50 mM Tris-HEPES (pH = 7.2), 5 mM EGTA, 5 mM EDTA, and 1 mM PMSF. The homogenates were centrifuged at 1500×*g* (human tissues) and 800×*g* (rat tissues) for 10 min, at 4 °C. Supernatants were collected and stored in the freezer at − 80 °C. The protein concentration was determined using the Pierce BCA protein assay kit (Thermo Fisher Scientific, USA).

Protein homogenates (30–70 µg) were mixed with 4 × Laemmli Sample Buffer (BioRad, USA; 1 × final concentration supplemented with 10% β-mercaptoethanol) and heated at 96 ℃ for 5 min. Proteins were separated by SDS-PAGE on polyacrylamide gels and electrophoresis was performed at 120 V, as previously described^7,24^. Proteins were transferred to PVDF membranes (BioRad, USA), and subsequently blocked for 1 h at room temperature in 5% BSA in TBS-T (20 mM Tris–HCl pH 7.6, 150 mM NaCl, 0.1% Tween 20). After three steps of washing, the membranes were incubated with specific primary antibodies overnight at 4 °C. The following primary rabbit antibodies reactive for humans and rats were used: TH (AB152, 1:1000, 10% gel; Merck, USA), DDC (AB1569, 1:500, 12.5% gel; Merck, USA), DBH (PA5-34,664, 1:1000, 10% gel; Invitrogen, USA), PNMT (LS-C482464, 1:1000, 15% gel; LifeSpan BioSciences, USA), COMT (PA5-51,523, 1:500, 15% gel; Invitrogen, USA), NET/SLC6A2 (Ab41559, 1:500, 10% gel; Abcam, UK), DAT/SLC6A3 (D6944, 1:1000, 10% gel; Sigma Aldrich, USA). After washing with TBS-T buffer, the membranes were incubated with anti-rabbit Immunoglobulins/HRP secondary antibody (P0217, 1:20,000; Dako, USA) for 1 h at room temperature. Membranes were developed using Amersham ECL Prime (GE Healthcare Life Science, USA) and the band intensity was quantified by densitometric analysis using the ChemiDoc MP, Imaging system (Bio-Rad, USA). To ensure the equal loading of proteins, membranes were probed for β-actin (Ab8226, 1:10,000; Abcam, UK) and specific secondary antibody (P0260, 1:20,000; Dako, USA). As positive controls, we used lysates from rat fetal brain (TH), rat adrenal gland (PNMT), rat maternal brain (NET), PC-12 cells (TH), SH-SY5Y cells (DDC, DBH, DAT), and HepG2 cells (DDC, COMT). Representative original immunoblots can be found in Supplementary Figs. S2 and S3.

### Statistical analysis

All statistical analyses were implemented in GraphPad Prism 9.3.1 software (GraphPad Software, Inc.). Expression results were first log2 transformed before analysis. Effect of trophoblast differentiation on catecholamine system expression was evaluated using paired t tests. Gene and protein expression results were assessed using Two-way ANOVA with Sidak's multiple comparisons test. Asterisks in the figures indicate significance levels: *p ≤ 0.05, **p ≤ 0.01, and ***p ≤ 0.001.

### Ethics approval and consent to participate

Experiments on human placentas were performed following the Declaration of Helsinki. Placental sample collection was obtained upon women's written informed consent and with the approval of the University Hospital Research Ethics Committee (201006 S15P). All procedures on rats were compliant with the ARRIVE guidelines and following the U.K. Animals (Scientific Procedures) Act, 1986 and associated guidelines of EU Directive 2010/63/EU for animal experiments. Experiments were approved by the Ethical Committee of the Faculty of Pharmacy in Hradec Kralove (MSMT-4312/2015–8).

## Results

### Differential expression of genes involved in catecholamine homeostasis in placental cells

**Figure 2:**
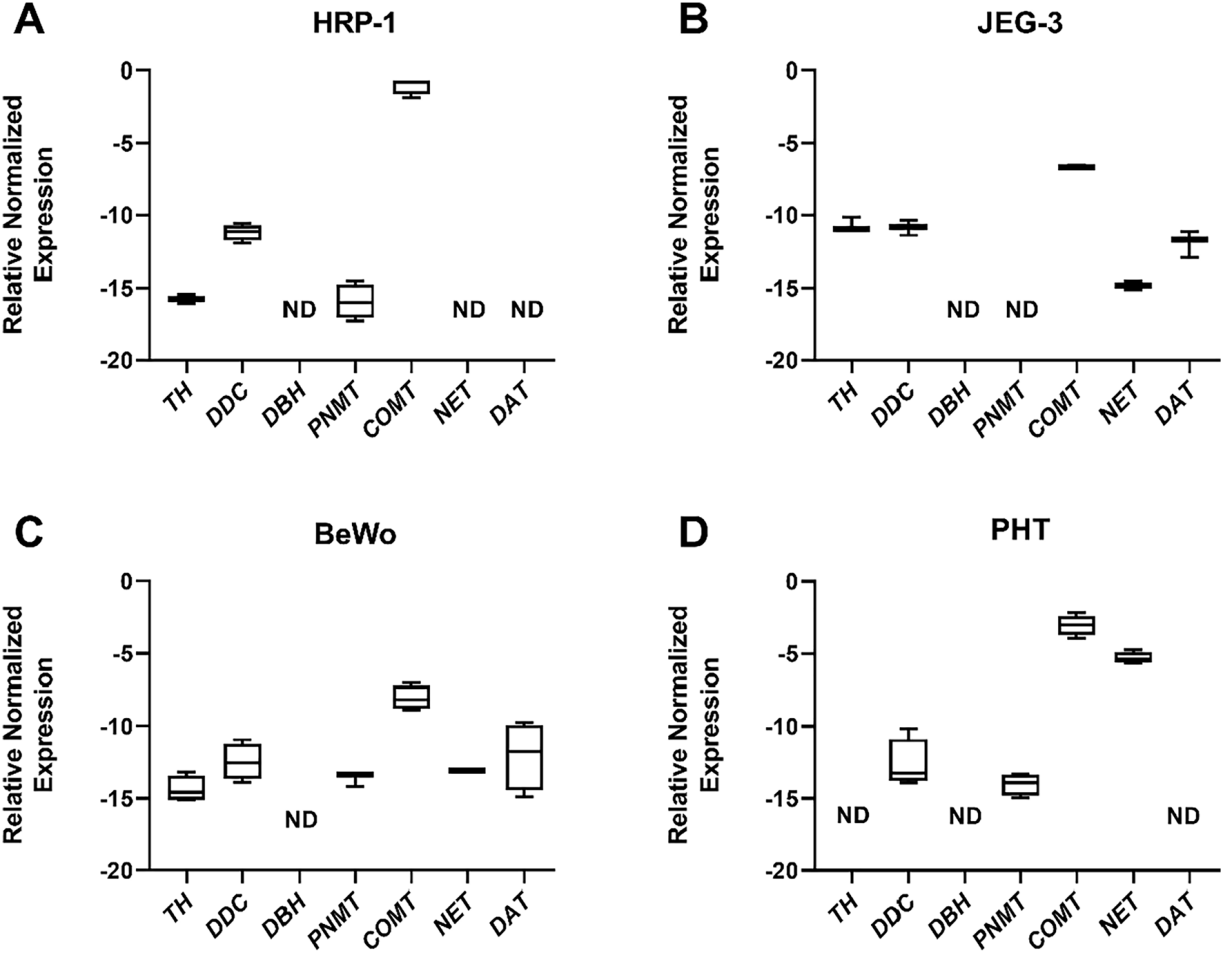
Gene expression of the catecholaminergic system in placental in vitro models. The expression of catecholamine synthesizing/degrading enzymes and transporters was investigated in the rat trophoendodermal cell line HRP-1 (**A**), human choriocarcinoma cells JEG-3 (**B**) and BeWo (**C**), and in primary trophoblast (PHT) cells isolated from human term placenta (**D**). Target gene expression was normalized against the geometric mean expression of *Gapdh* and *Ywhaz* (**A**) or *GAPDH* and *B2M* (**B**–**D**). Data are shown as Tukey boxplots (1.5-times IQR) after log2 transformation; n = 4. ND – not detected.

The expression of enzymes/transporters involved in catecholamine synthesis, metabolism, and transport was investigated in the rat trophoendodermal cell line HRP-1, human choriocarcinoma cells BeWo and JEG-3, and in PHT cells isolated from human term placenta. We observed significant differences between these commonly used placental models in expressing several enzymes and transporters (Fig. 2). Specifically, BeWo and JEG-3 cells showed comparable profiles of expression, however HRP-1 and PHT cells were deficient in the expression of several components. The rat trophoendodermal cell line (Fig. 2A) and the choriocarcinoma-derived cells (Fig. 2B,C) revealed expression of *TH* and *DAT*, which were not expressed in PHT cells (Fig. 2D). On the other hand, JEG-3 cells lack the expression of *PNMT*, observed in other cell models (Fig. 2B), whereas HRP-1 cells did not express any of the catecholamine transporters (Fig. 2A). Notably, *DBH* was not expressed in any of the placental cells.

**Figure 3:**
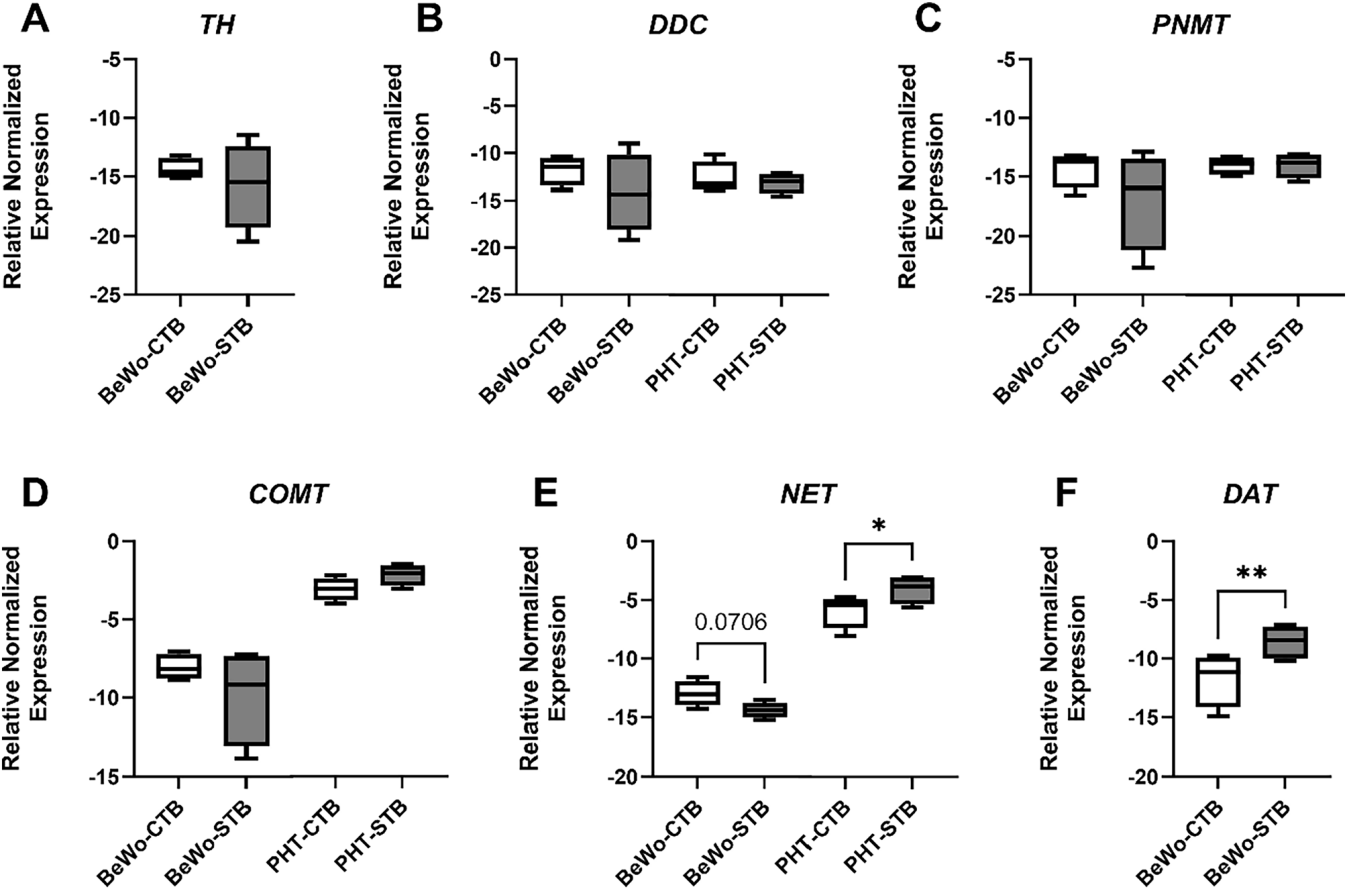
Effect of trophoblast differentiation on the expression of genes involved in placental catecholamine homeostasis. Differentiation studies were addressed in BeWo human choriocarcinoma cell line and isolated primary trophoblast cells from the human term placenta (PHT). BeWo cells' differentiation was induced by incubation with 20 µM forskolin for 24 h, whereas in PHT cells, this was achieved spontaneously during a 72-h culture period, with a daily change of medium. The CTB and STB stage represent the undifferentiated and differentiated cells, respectively. Target gene expression was normalized against the geometric mean expression of *GAPDH* and *B2M.* Data are shown as Tukey boxplots (1.5-times IQR) after log2 transformation; n = 4. Statistical analysis between the two cell stages was performed using paired t tests. *p ≤ 0.05, **p ≤ 0.01.

Additionally, the effect of trophoblast differentiation on pathway expression was examined in BeWo and PHT cells. These cells are the only placental cell models to undergo trophoblast differentiation spontaneously (PHT cells)^27^ or upon stimulation with syncytializing agents such as forskolin (BeWo)^28^. We observed that the expression of *TH*, *DDC*, *PNMT*, and *COMT*, is not regulated by the transition to differentiated STB (Fig. 3A–D). On the other hand, *NET* expression showed significant upregulation by the spontaneous differentiation in vitro; this was not true for forskolin-induced BeWo differentiation (Fig. 3E), suggesting differential mechanisms involved in regulating these genes compared to PHT cells. Moreover, *DAT,* which is only expressed in BeWo cells, showed significant upregulation in response to forskolin treatment (Fig. 3F).

### Gene and protein expression analysis of catecholamine system in human first-trimester and term placenta

**Figure 4:**
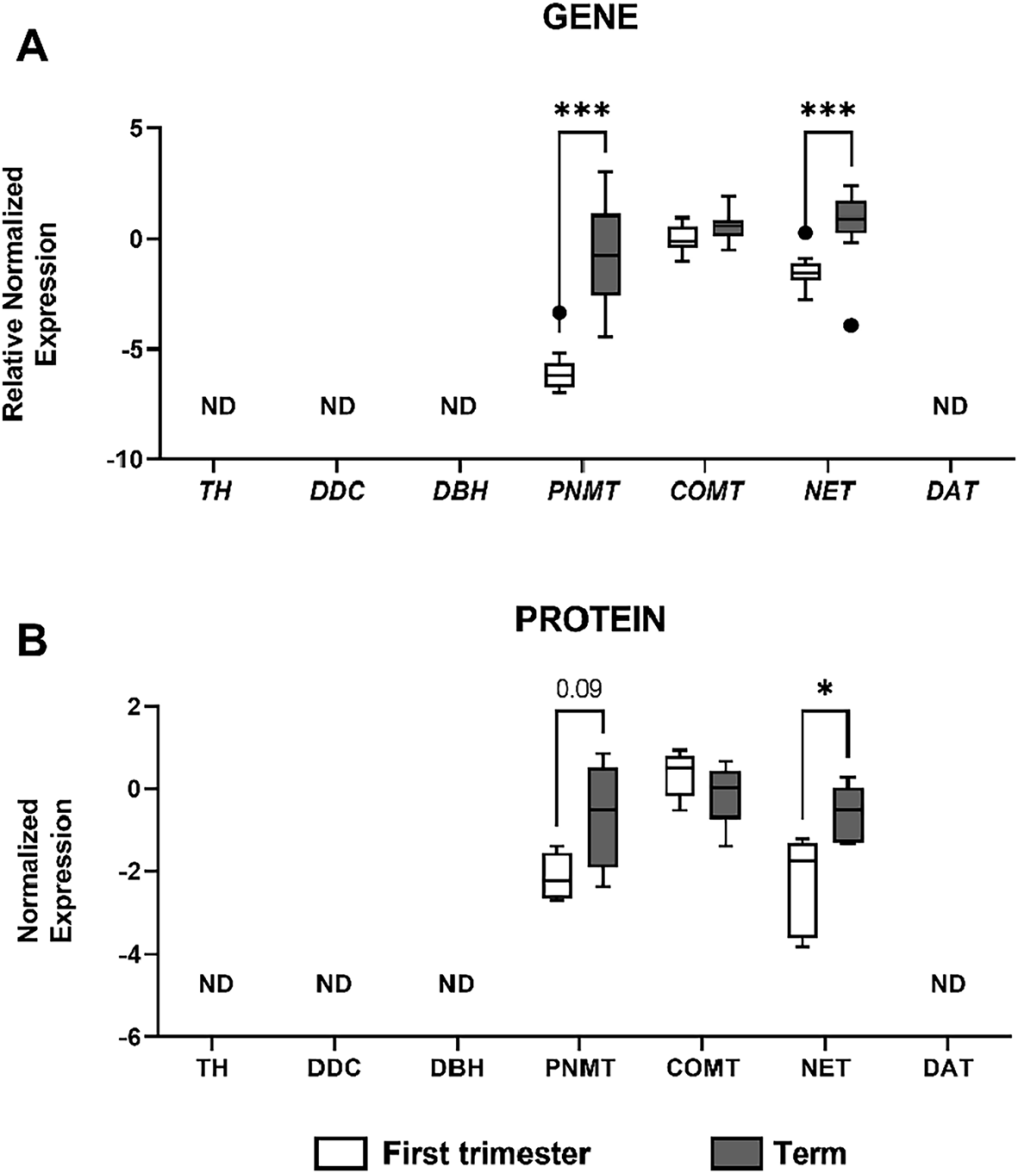
The expression of catecholamine system in the human placenta during gestation. qPCR and western blot analysis were employed to investigate gene (**A**) and protein (**B**) expression of catecholamine synthesizing and degrading enzymes, and transporters. Target gene expression was normalized against the geometric mean expression of *GAPDH* and *B2M*; n = 13 first trimester/28 term. Protein expression was normalized to β-actin as a loading control; n = 5 for each. Data are shown as Tukey boxplots (1.5-times IQR); after log2 transformation (**A**). Statistical analysis was performed using Two-way ANOVA with Sidak's multiple comparisons test. *p ≤ 0.05, ***p ≤ 0.001. ND – not detected.

To investigate how advancing gestation affects the expression of enzymes and transporters involved in placental homeostasis of catecholamines, we performed qPCR and western blot analysis in a healthy pregnancy cohort of first trimester (after elective interruption of pregnancy) and term (at delivery) tissues. Out of 7 genes tested, only *PNMT*, *COMT*, and *NET* were expressed in the human placenta (Fig. 4A). This was also reflected at the protein level where we only detected the expression of PNMT, COMT, and NET (Fig. 4B). Importantly, the expression of these genes was affected as a function of gestation age. Specifically, gene and protein expression of NET and PNMT revealed higher expression as pregnancy proceeded to term.

Within the term placenta cohort, it is important to note that we included an equal number of samples from both cesarean section and spontaneous birth deliveries for gene expression studies. We conducted tests to assess whether gene expression differed between the two groups and found no significant differences (see Supplementary Fig. S4). However, due to the limited sample size, we were unable to conduct similar analyses for protein expression.

### Gene and protein expression analysis of catecholamine system in the rat placenta during gestation

**Figure 5:**
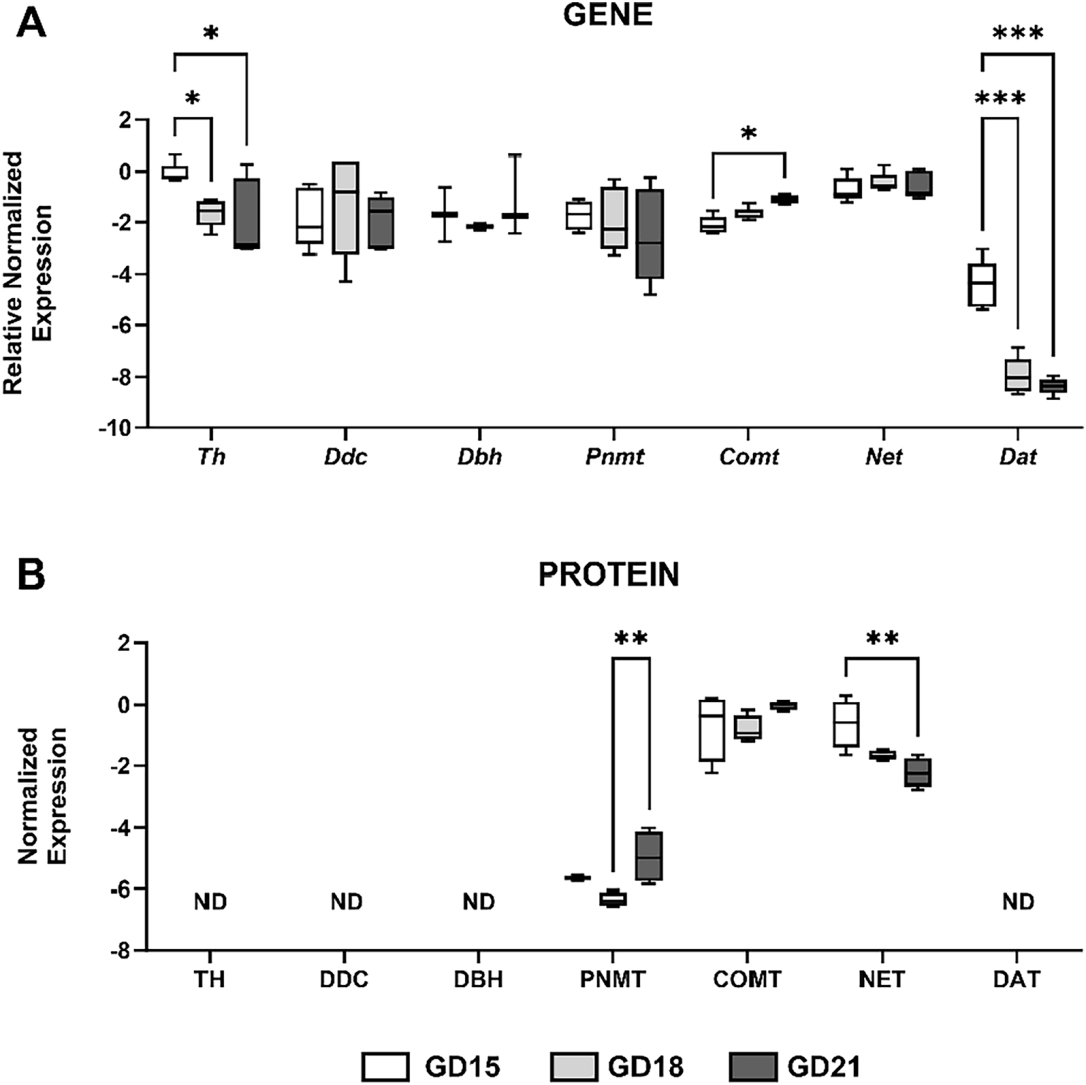
The expression of catecholamine system in the rat placenta during gestation. qPCR and western blot analysis were employed to investigate gene (**A**) and protein (**B**) expression of catecholamine synthesizing and degrading enzymes, and transporters. The experiments were conducted in placentas from gestation day (GD) 15, 18, and 21. Target gene expression was normalized against the geometric mean expression of *Gapdh* and *Ywhaz*; n = 5 for each. Protein expression was normalized to β-actin as a loading control; n = 4 for each. Data are shown as Tukey boxplots (1.5-times IQR); after log2 transformation (**A**). Statistical analysis was performed using Two-way ANOVA with Sidak's multiple comparisons test. *p ≤ 0.05, **p ≤ 0.01, ***p ≤ 0.001. ND – not detected.

To study the changes in catecholamine pathways from mid- to late pregnancy, we employed the rat model and analyzed the gene and protein expression of the 7 selected enzymes/transporters in the placentas from GD 15, 18, and 21. Compared to human tissue, rat placenta expressed all the enzymes investigated at the mRNA level (Fig. 5A). We observed that *Th* and *Dat* gene expression decreased towards term, while *Comt* was upregulated with advancing gestation (Fig. 5A). Nonetheless, at protein level we only detected the expression of PNMT, COMT, and NET (Fig. 5B). Furthermore, NET protein expression was downregulated as the pregnancy progresses from GD15 to GD21 (Fig. 5B).

### Expression of the catecholamine genes in rat fetal organs

**Figure 6:**
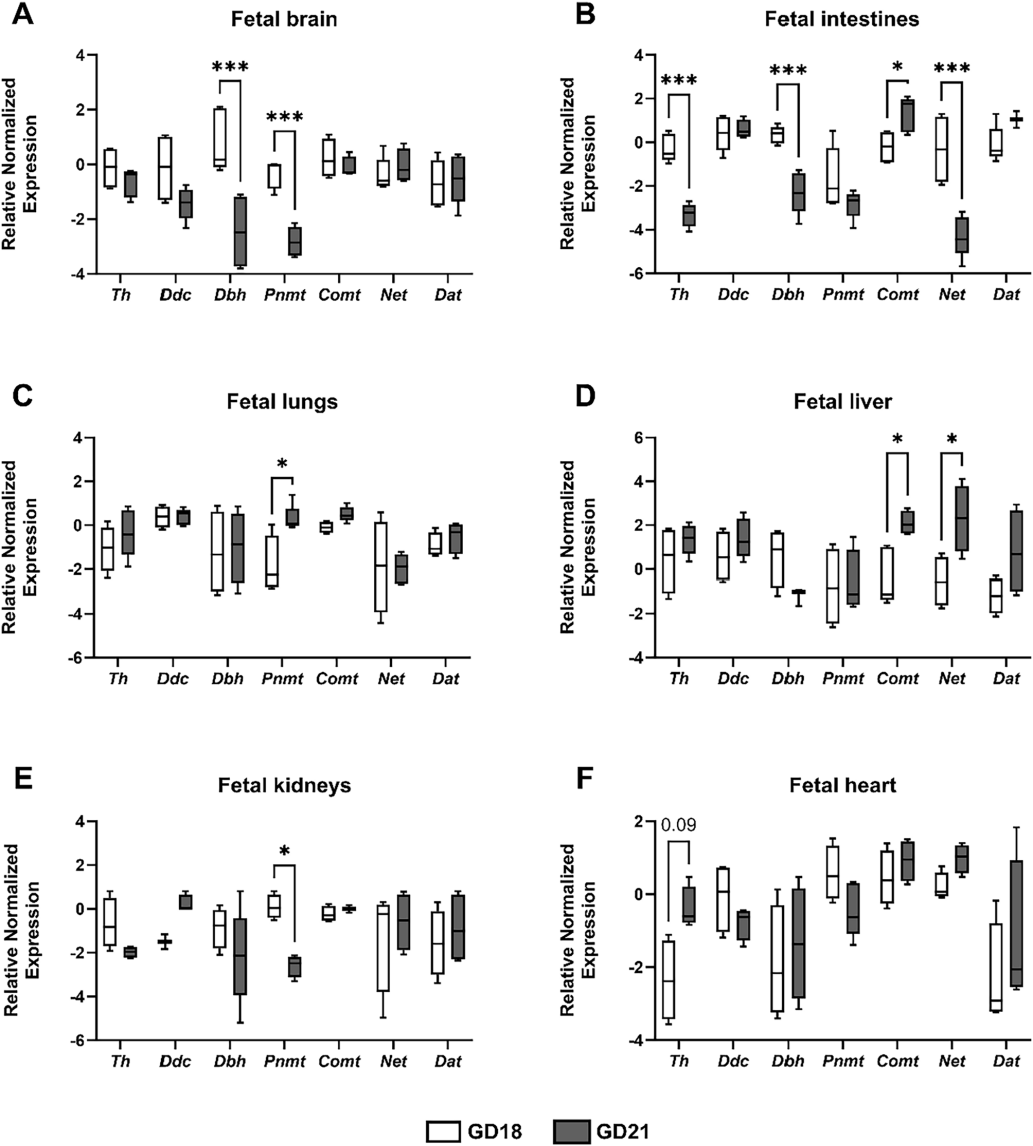
The expression of catecholamine system in the rat fetal organs. Gene expression of catecholamine synthesizing and degrading enzymes, and transporters was evaluated in fetal brain (**A**), intestines (**B**), lungs (**C**), liver (**D**), kidneys (**E**), and heart (**F**). The experiments were conducted in fetuses from gestation day (GD) 18 and 21. Target gene expression was normalized against the geometric mean expression of *Gapdh* and *Ywhaz*. Data are shown as Tukey boxplots (1.5-times IQR) after log2 transformation; n ≥ 4 for each. Statistical analysis was performed using Two-way ANOVA with Sidak's multiple comparisons test. *p ≤ 0.05, ***p ≤ 0.001.

Next, we assessed the fetal capacity to participate in catecholamine synthesis, metabolism, and transport. Rat fetal brain, intestines, lungs, liver, kidneys, and heart were examined for the gene expression of key enzymes/transporters. All fetal tissues examined expressed the selection of genes tested, and certain developmental expression patterns were observed (Fig. 6). From the enzymes involved in catecholamine synthesis from tyrosine, *Th* expression at term was downregulated in fetal intestines and upregulated in fetal heart (Fig. 6B,F). On the other hand, *Dbh* was downregulated in both fetal brain and intestines (Fig. 6A,B). *Pnmt* was the gene most substantially affected when comparing GD18 and 21, showing downregulation during gestation in fetal brain and kidneys (Fig. 6A,E), and upregulation in fetal lungs (Fig. 6C). The metabolizing enzyme *Comt* showed higher expression in GD21 in both fetal intestines and liver (Fig. 6B,D). On the other hand, *Net* expression during gestation had differing patterns in fetal intestines and liver, with the intestines showing higher expression at GD18 (Fig. 6B), whereas the liver at GD21 (Fig. 6D).

## Discussion

In this study, we used several placental cell models, to identify components of cellular catecholamine handling associated with the trophoblast cells. Next, we determined the effect of advancing gestation on the placental catecholamine system in humans and rats. Lastly, we detected the expression of catecholamine pathway genes in rat fetal organs, revealing distinct developmental expression patterns.

In vitro cell-based models are frequently used to investigate placental physiology and pathology; however the cellular repertoire of enzymes and transporters necessary for dopamine and norepinephrine synthesis, metabolism, and transport remain unclear. Here, we show that while these cell lines share a trophoblastic phenotype, their catecholamine system transcriptional profile differs largely. Additionally, although dopamine and norepinephrine regulate the production of hormones such as human placental lactogen and chorionic gonadotropin^29,30^, our findings from BeWo and primary trophoblast cells show that the expression of enzymes involved in catecholamine homeostasis is not regulated by trophoblast syncytium formation. It's crucial to highlight that while PHT cells exhibit similarities to overall tissue expression patterns, this does not imply a direct comparison of a compartment-specific cell line to the whole placenta.

Interestingly, while the choriocarcinoma-derived cells express *TH*, the rate-limiting enzyme in dopamine synthesis, PHT cells do not. Likewise, BeWo and JEG-3 cells show DAT expression at both mRNA, protein, and functional levels, which is not seen in other in vitro models^31^. This observation is noteworthy given their isolation from brain metastatic choriocarcinoma^32,33^. In a similar manner, the HRP-1 cells lack several crucial genes related to the catecholamine transport system. We have recently reported that the HRP-1 cells differ significantly in functional features of catecholamine transport, thereby discouraging their use in catecholamine research^25^. Together, these results from both human placenta primary cells and placenta-derived cell lines emphasize the importance of careful selection of the right in vitro model for studying the metabolism and transport of catecholamines in the placenta.

Understanding the synthesis, metabolism, and transport of dopamine, norepinephrine, and epinephrine in both the placenta and fetal brain is crucial for unravelling the complex mechanisms underlying neurodevelopment at the placenta-brain axis. With the recognition of the placenta as a source of serotonin during the prenatal period^8^, possibilities arose that it may also constitute a dopaminergic and noradrenergic organ; however, the dynamics of these systems in the placenta are less characterized. In the context of the human placenta, our research uncovered the presence of only PNMT, COMT, and NET at both the mRNA and protein levels. In contrast, parallel research in the rat placenta revealed a more extensive spectrum of gene expression encompassing all elements of the catecholamine pathway. However, protein expression profiles in rat placenta mirrored those in humans, with only PNMT, COMT, and NET detected. Notably, the absence of protein expression for TH, DDC, and DBH was observed in both human and rat placenta. This contradicts prior research, which reported DDC expression in the human placenta, however, suggesting the existence of an inactive enzyme form^34^. Additionally, older studies indicated the presence of TH and DBH expression in the placenta, primary trophoblast cultures, and trophoblast cell lines^35^, adding a layer of complexity to our current understanding of catecholamine pathway dynamics in placental tissues. Future studies should focus on investigations aiming to unravel the protein functions of the enzymes and transporters identified within the catecholamine pathway in both human and rodent placenta.

Interestingly, our observations revealed an upregulation of PNMT expression during gestation in both human and rat placenta, suggesting a conserved regulatory mechanism. Corresponding studies in humans showed that as gestation progresses towards term, the fetal circulation displays a significant increase in norepinephrine and epinephrine levels^36^. This could be a result of upregulated PNMT expression which might contribute to increased placental output of norepinephrine and epinephrine to the developing fetus. In addition, upregulated norepinephrine could also be attributed to increased transplacental transport of norepinephrine via NET. Our data in the human placenta support this speculation, revealing an upregulation of NET expression at term. However, our observations in rats indicate the opposite trend. These findings highlight the complexity of species-specific regulatory pathways and prompt further exploration into the complex dynamics governing placental physiology.

In the landscape of fetal brain development, the synthesis, metabolism, and transport of catecholamines play pivotal roles in orchestrating neurodevelopmental processes. Our findings revealed a comprehensive expression of all enzymes and transporters throughout gestation, which is in line with previous findings indicating region-specific expression patterns^37,38^. We showed that most genes exhibit stable expression from mid- to term gestation. Notably, we observed a downregulation of *Dbh* and *Pnmt* at term, suggesting regulatory mechanism involved. Studies have shown that the administration of tyrosine to pregnant rats and mice results in increased levels of tyrosine, dopamine, and norepinephrine in the fetal brain^39–41^, indicative of a fully functional enzymatic and transport machinery in the fetoplacental unit. This phenomenon occurs around midgestation and coincides with the arrival of TH-positive axons in the lateral ganglionic eminence^40,41^; thus, highlighting the developmental timing of the fetal brain catecholamine system's synthesizing capacity. Despite these early activities, the most dramatic increase in functionality is observed postnatally^37^. Collectively, these findings underscore the importance of catecholamine metabolism in shaping neurodevelopmental outcomes.

In general, the concentrations of catecholamines in fetal circulation are low during pregnancy, with levels substantially increasing at birth. This is considered a critical process for cardiovascular, pulmonary, metabolic, and endocrine adaptations during postnatal life^42^. Nonetheless, catecholamines play important roles during embryonic development; thus, tightly regulated intrinsic catecholamine homeostasis is critical. In line with previous reports^43,44^, intrinsic cardiac catecholamine biosynthesis was evidenced by *Th*, *Dbh*, *Pnmt* expression in the fetal heart. Supportive of this are findings that, when perfused with tyrosine, the human fetal heart synthesizes dopamine and norepinephrine^45^. Our observations of increased *Th* expression in the rat fetal heart during gestation correspond well with previously reported increases in epinephrine concentrations in the fetal heart towards term^43^. Upregulated catecholamine synthesis in late gestation likely involves the ventricular components of the cardiac conduction system^43^, as norepinephrine and epinephrine are involved in maintaining the heart rate^46^ and are essential for fetal development and postnatal adaptation^47^.

Current evidence highlights the expression of DDC in adult rat liver and kidney^48^. Moreover, dopamine has been found as the principal catecholamine excreted in healthy human urine, with predominant renal origin^49^. Interestingly, amniotic fluid dopamine levels increase dramatically towards term^36^. As fetal urine is a significant contributor to the amniotic fluid, it is likely that the fetal kidneys are responsible for this rise; this hypothesis is supported by our findings of upregulation of renal *Ddc* toward term. However, it cannot be excluded that the fetal lungs also contribute to amniotic fluid dopamine, a mechanism supposedly important for lung liquid reabsorption at birth^50^. The lungs are also a site of PNMT expression and activity^51^. Here, we observed that the expression of *Pnmt* in fetal lungs significantly increases at term, which is in line with a previous study showing upregulated PNMT activity in newborns, likely as a process of lung maturation^51^.

The catecholamine metabolizing and transport proteins play an important role in catecholamine disposition and effects. Although their distribution is described in adults, their expression in fetal tissues has not been fully characterized. Current evidence highlights that, outside of the CNS, COMT is expressed in the liver, kidney, and intestines, with the liver and kidneys exhibiting the highest COMT activity^52^. Here we show that the fetal liver and intestines are associated with upregulated *Comt* at term; thus, this regulatory mechanism may represent potential maturation of the organ for life after birth. Similarly, we show that while all tissues expressed NET and DAT, expression of NET is significantly reduced in the fetal intestines, whereas in the fetal liver, it is upregulated.

Integrating findings from human and animal models enhances our understanding of the dynamic regulatory mechanisms that underlie catecholamine dynamics during pregnancy. We identified similar patterns in both species across gestational ages, suggesting conserved molecular mechanisms and potentially shedding light on shared biological processes influencing placental development. However, it is essential to acknowledge that the observed disparities between mRNA and protein levels highlight the importance of a more in-depth investigation into the functional implications of these observations. Studies have shown that catecholamines may also represent important signals secreted by the fetoplacental unit in pregnancy disorders associated with placental dysfunction and impaired blood flow, such as preeclampsia and intrauterine growth restriction^10,35^. Additionally, drug use, like cocaine, can disrupt dopaminergic signaling in the developing fetal brain^53^. This disturbance in dopamine and other neurotransmitters is linked to various conditions, including schizophrenia, autism, ADHD, negative temperament, and cognitive delays^54–57^. Therefore, further research in this field holds promise for elucidating the implications of altered fetoplacental catecholamine regulation in pregnancy-related disorders and neurodevelopmental abnormalities.

## Conclusions

In conclusion, our study comprehensively explores catecholamine dynamics in the fetoplacental unit, using diverse in vitro models and gestational comparisons. The identification of distinct transcriptional profiles in various placenta-derived cell lines underscores the importance of careful selection of an in vitro experimental model. Moreover, integrating findings from humans and rats enhances our understanding of conserved regulatory mechanisms, shedding light on shared biological processes influencing placental development across gestation. This insight establishes a foundation for further investigations into pregnancy-related disorders and neurodevelopmental abnormalities.

### Supplementary Information

Supplementary Information.

## Data Availability

All data generated or analyzed during this study are included in this published article (and its supplementary information files).

## Abbreviations

COMT: Catechol-o-methyltransferase
CTB: Cytotrophoblast
DAT: Dopamine transporter
DBH: Dopamine β-hydroxylase
DDC: Dopa decarboxylase
hCG: Human chorionic gonadotropin
MAO: Monoamine oxidase
NET: Norepinephrine transporter
PNMT: Phenylethanolamine N-methyltransferase
PHT: Primary trophoblast
STB: Syncytiotrophoblast
TH: Tyrosine hydroxylase
