# Supplementary material for: Developmental expression of catecholamine system in the human placenta and rat fetoplacental unit
Source: Sci Rep. 2024 Mar 23;14:6948. doi: 10.1038/s41598-024-57481-5 (PMC10960862; doi:10.1038/s41598-024-57481-5)
Supplement: Supplementary file 1 — Supplementary Information. [file 41598_2024_57481_MOESM1_ESM.docx]

**ADDITIONAL FILES**

**Table S1.** Clinical characteristics of pregnancies included in the study.

|  | First trimester (n = 13) | Term (n = 28) |
| --- | --- | --- |
| Maternal age (years) | 27.91 ± 8.04 | 32.74 ± 5.45 |
| Gestational age (weeks) | 9.62 ± 1.19 | 38.06 ± 7.52 |
| Maternal BMI at delivery (kg/m2) | 24.48 ± 3.31 | 28.21 ± 4.43 |
| Labor (spontaneous:c-section) | NA | 14:14 |
| Birth weight (kg) | NA | 3.47 ± 0.42 |
| Birth height (cm) | NA | 49.93 ± 1.98 |
| Fetal sex (male:female) | NA | 19:9 |

*All are expressed as mean ± SD. NA - not applicable.*

**Figure S1.** **Comparison of human chorionic gonadotropin (hCG) release in undifferentiated (CTB) and differentiated (STB) cells.** hCG levels were measured in the media of BeWo cells (A) and primary trophoblast (PHT) cells isolated from human term placenta (B) using an ELISA kit. Data are shown as Tukey boxplots (1.5-times IQR); n = 4.


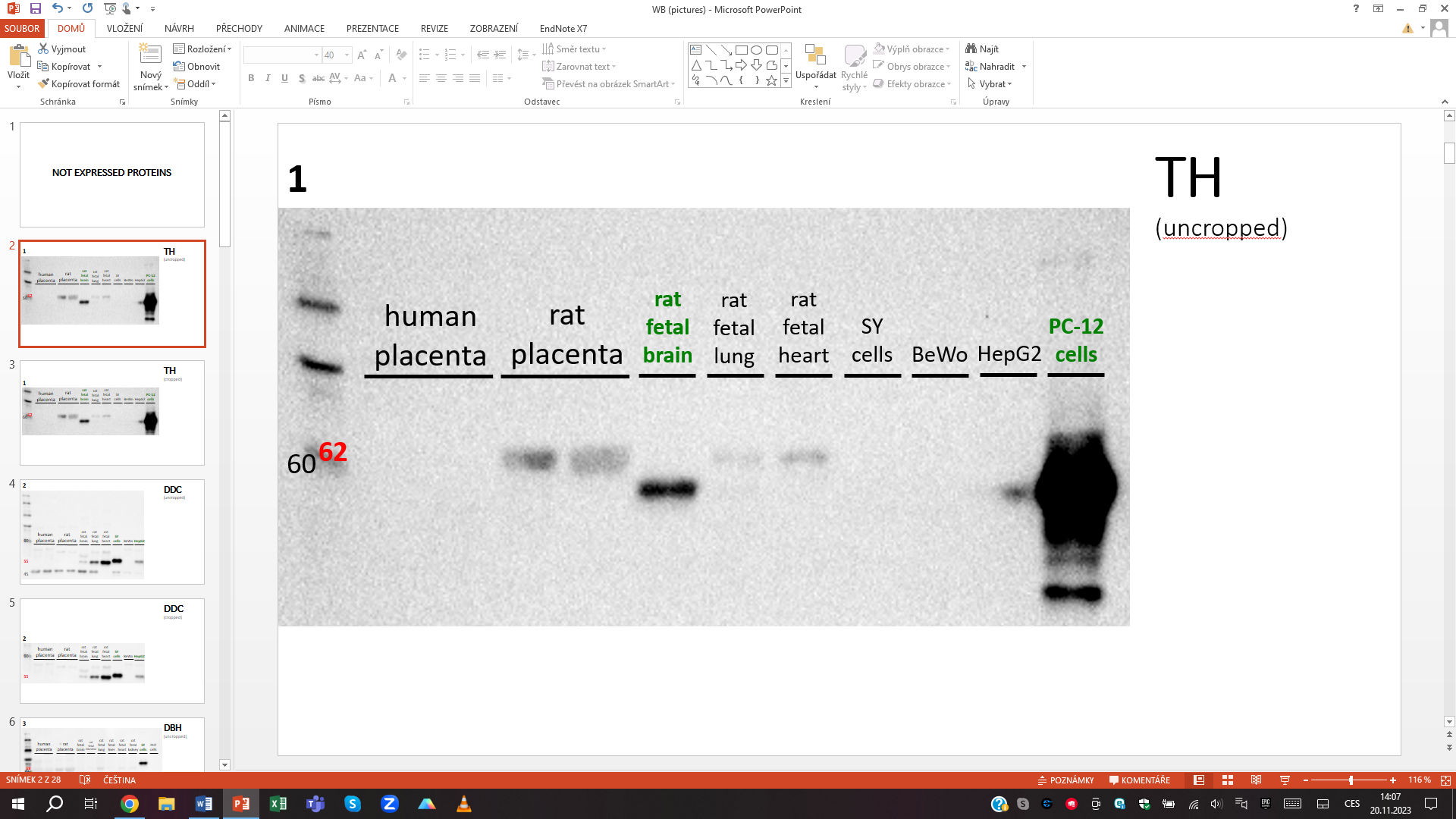

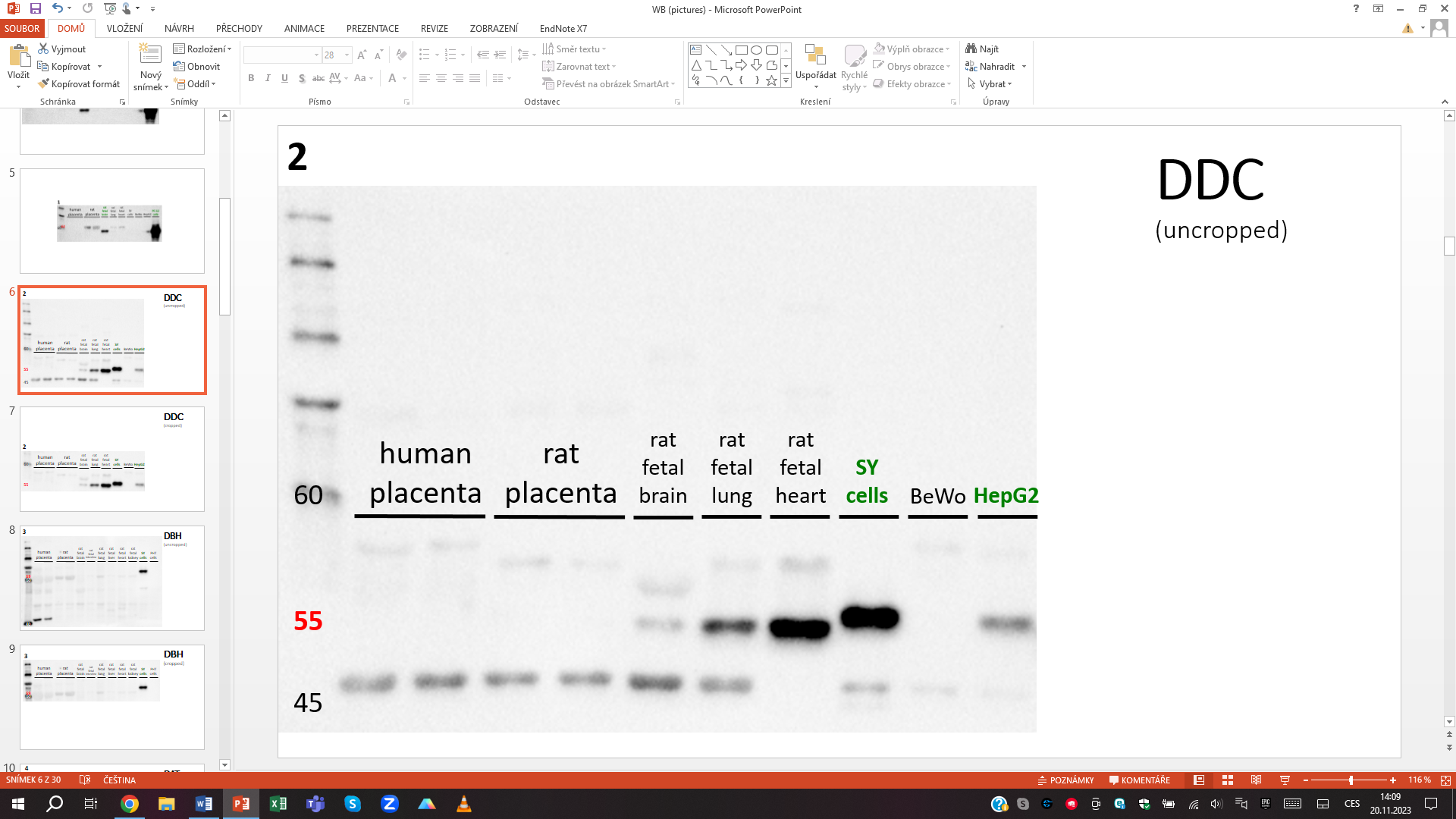

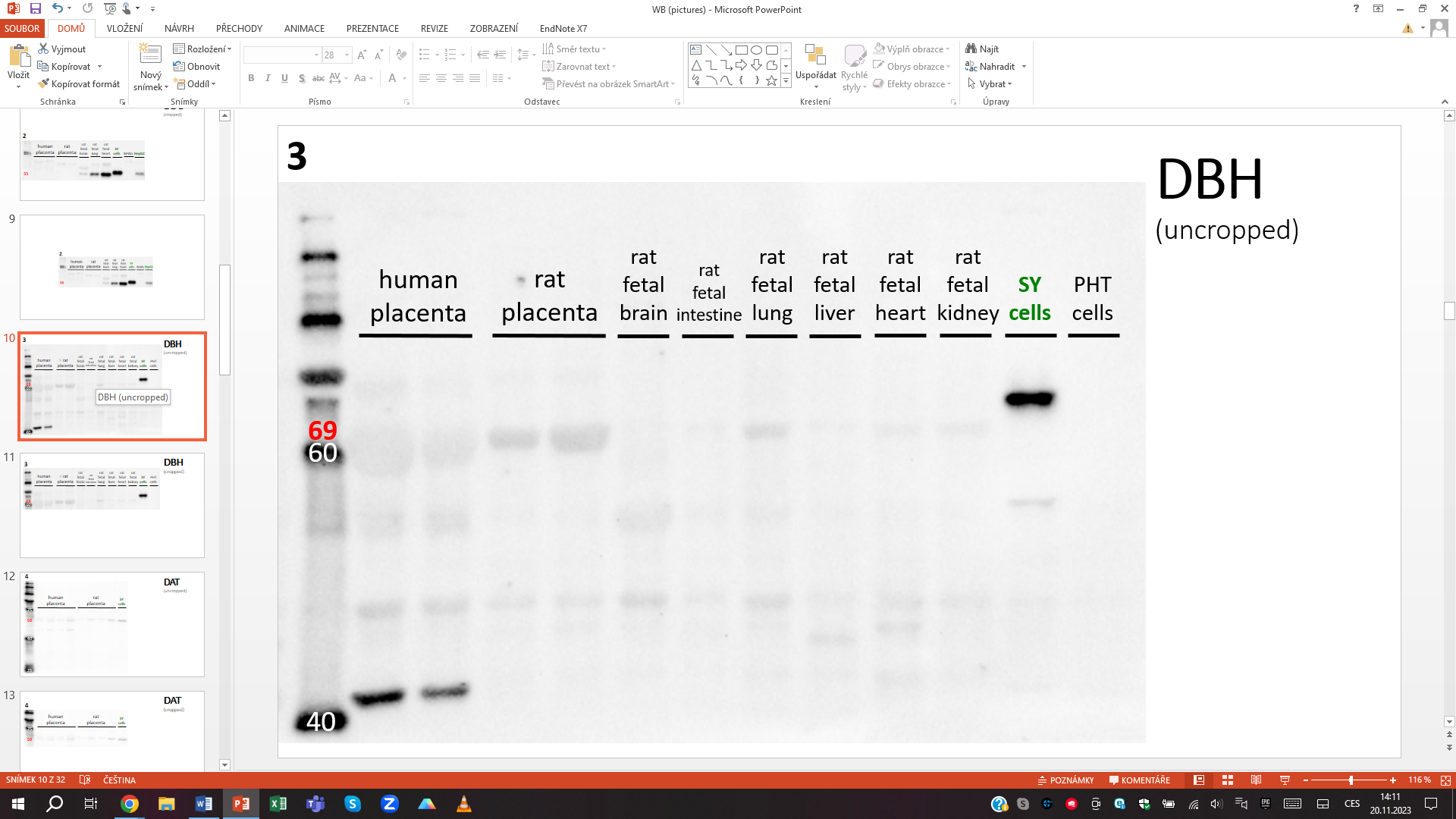

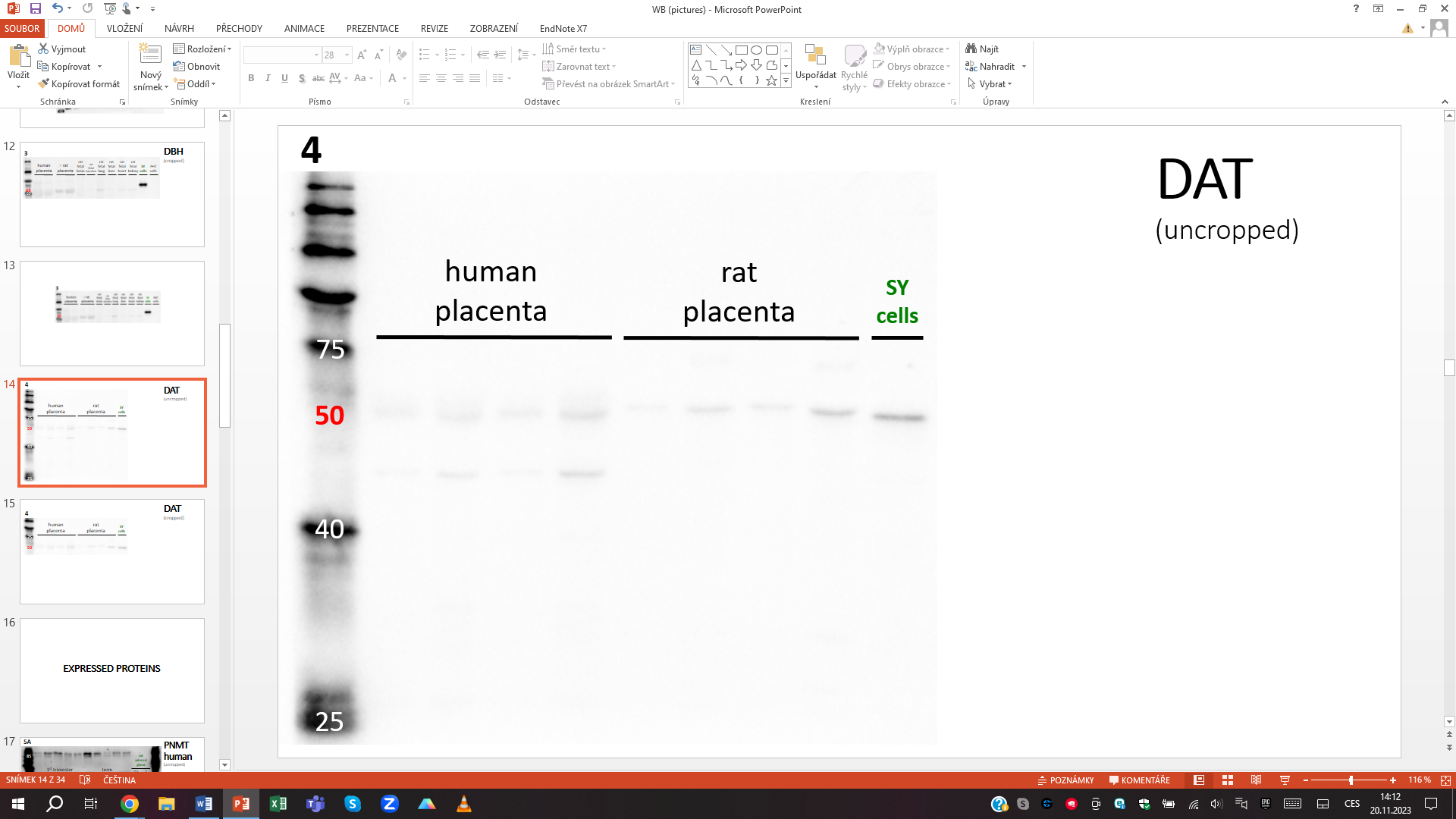


**Figure S2. Raw (uncropped) representative immunoblots from Western Blot analysis of unexpressed proteins.** Target proteins were analysed in human and rat placentas (TH – 1, DDC – 2, DBH – 3, DAT - 4). Rat fetal brain, PC-12 cells, HepG2 cells, and SY (SH-SY5Y) cells were used as positive controls and are indicated in green labelling for each target protein. Target protein size (kDa) is indicated in red label. No stripping of the initial antibody was performed before proceeding with the loading control.


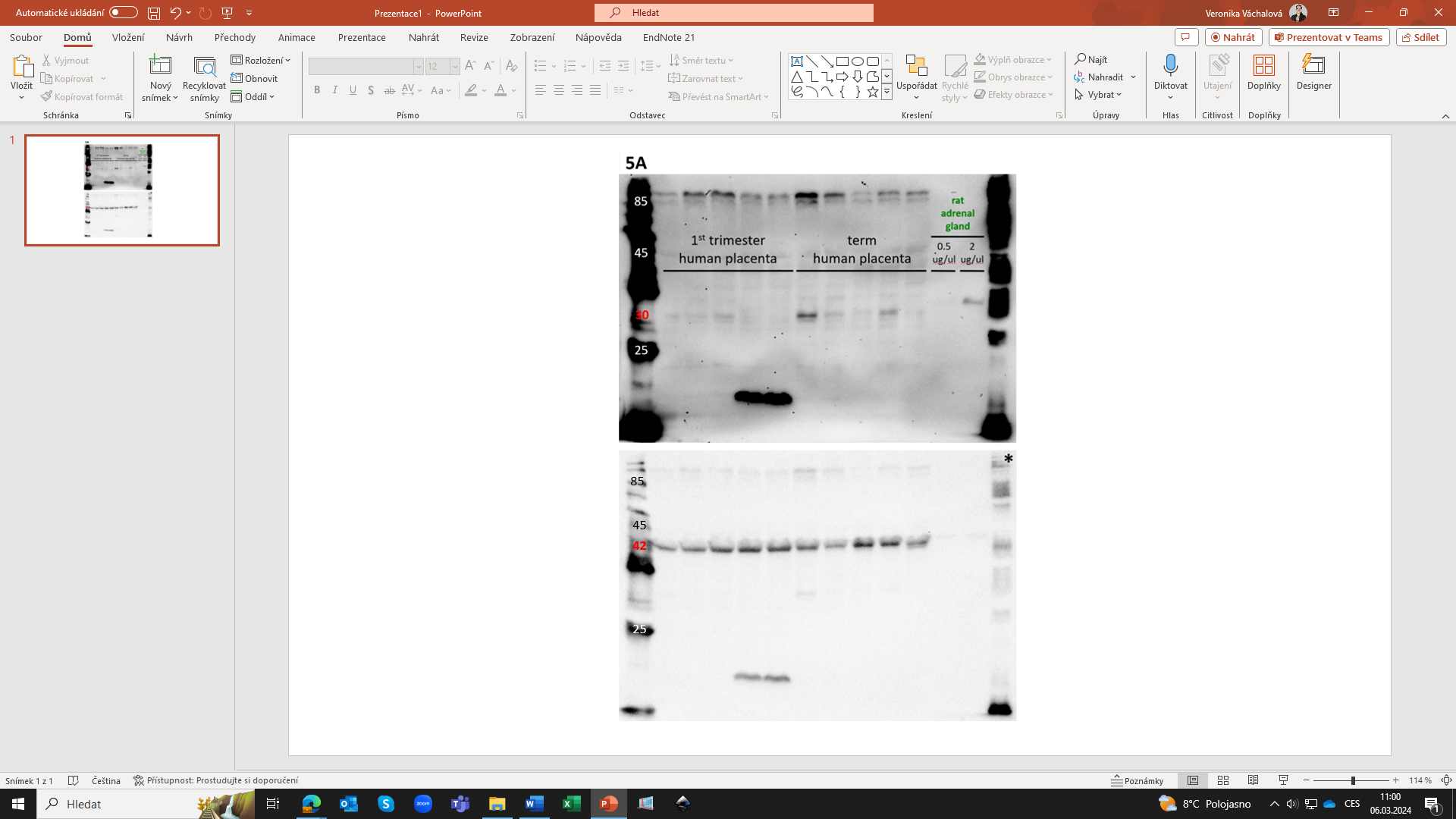

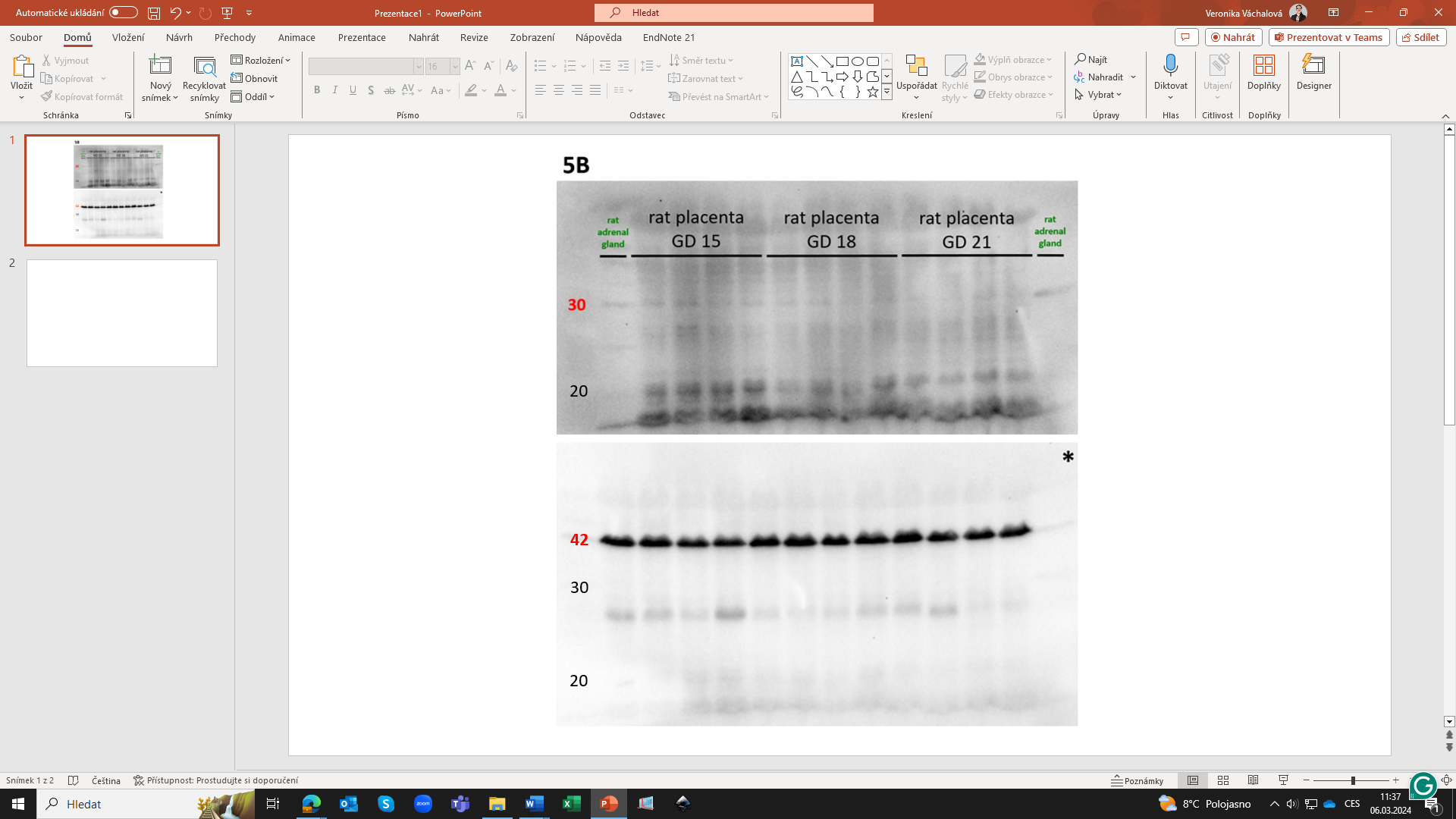


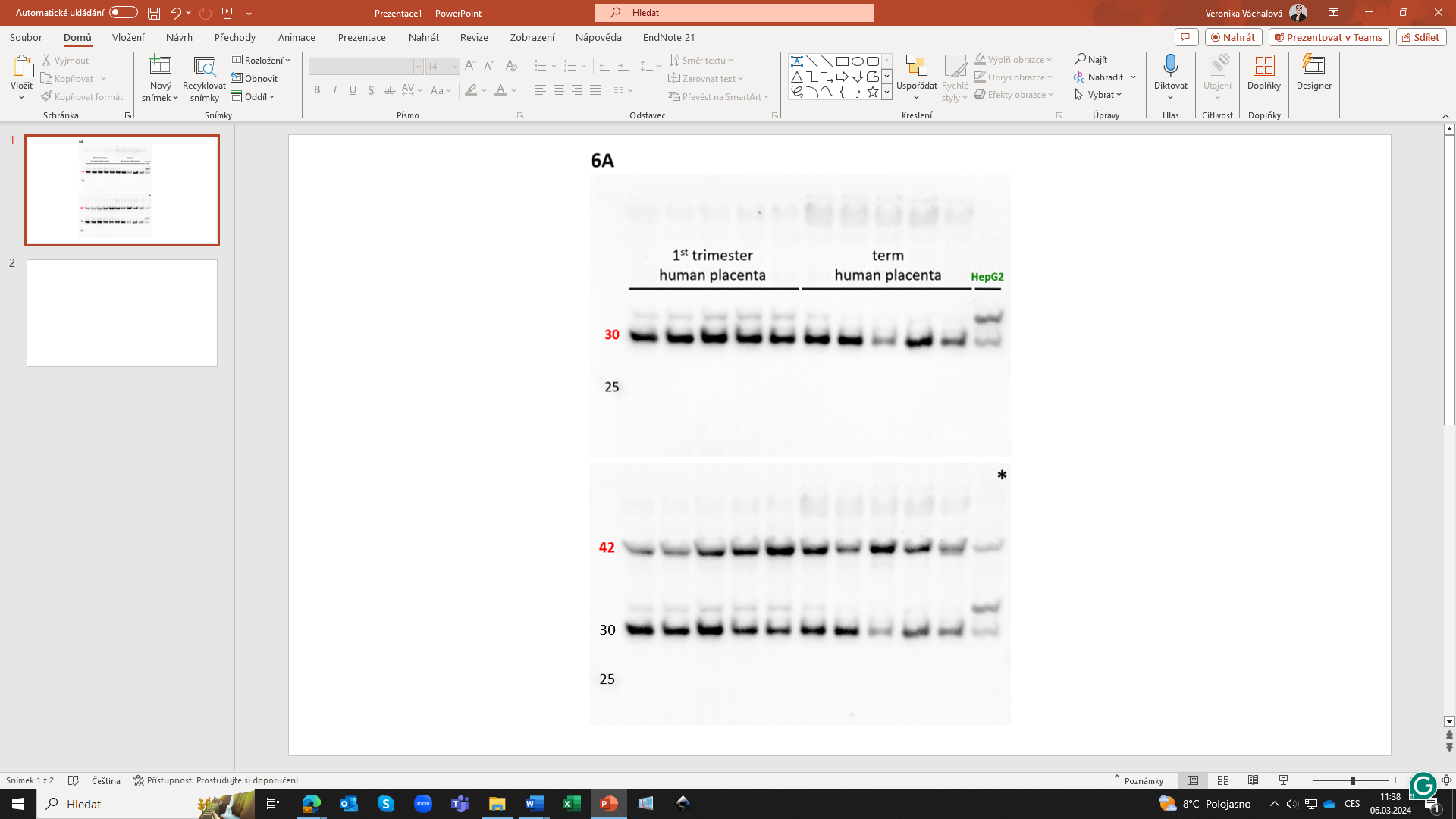

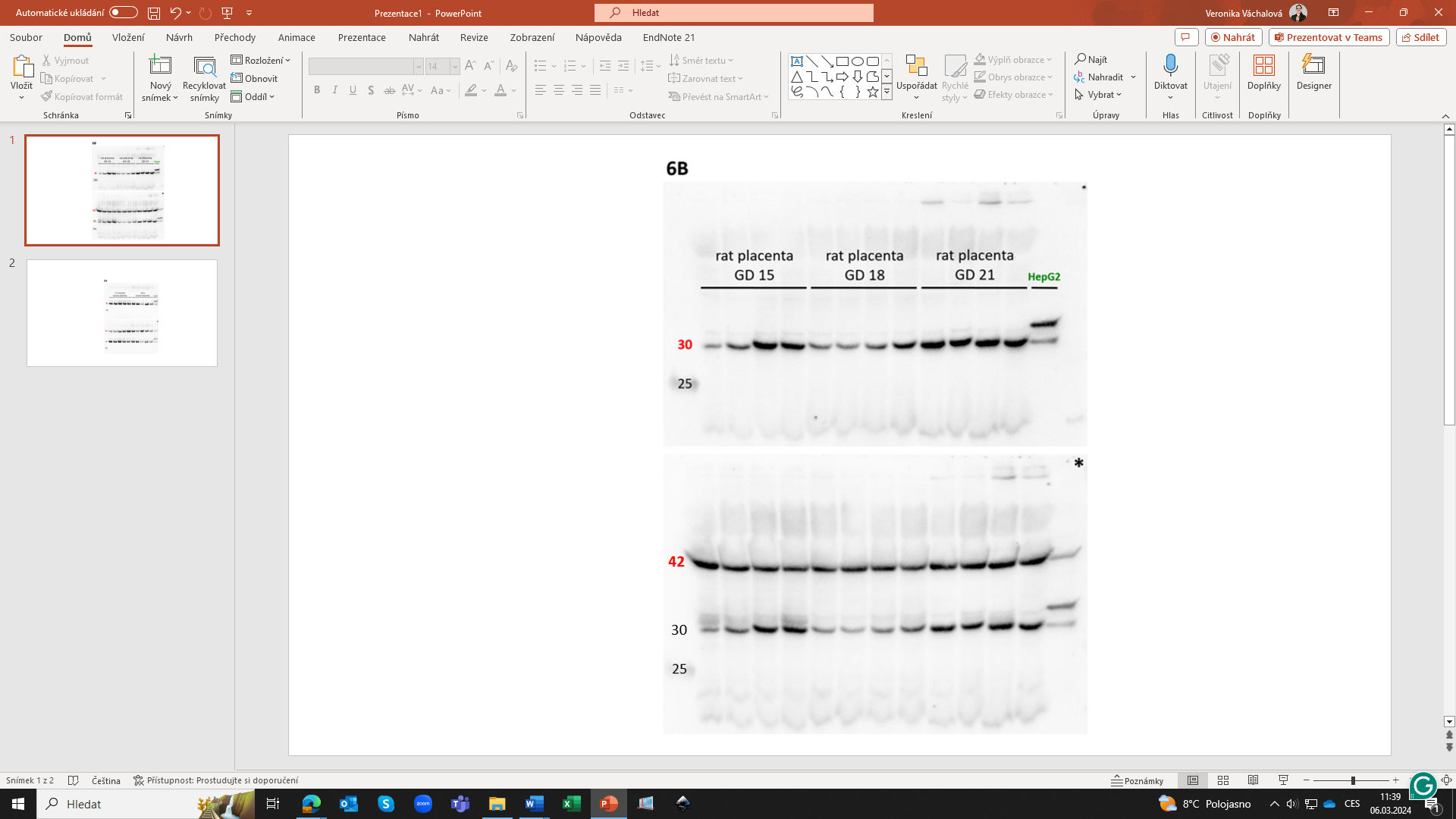


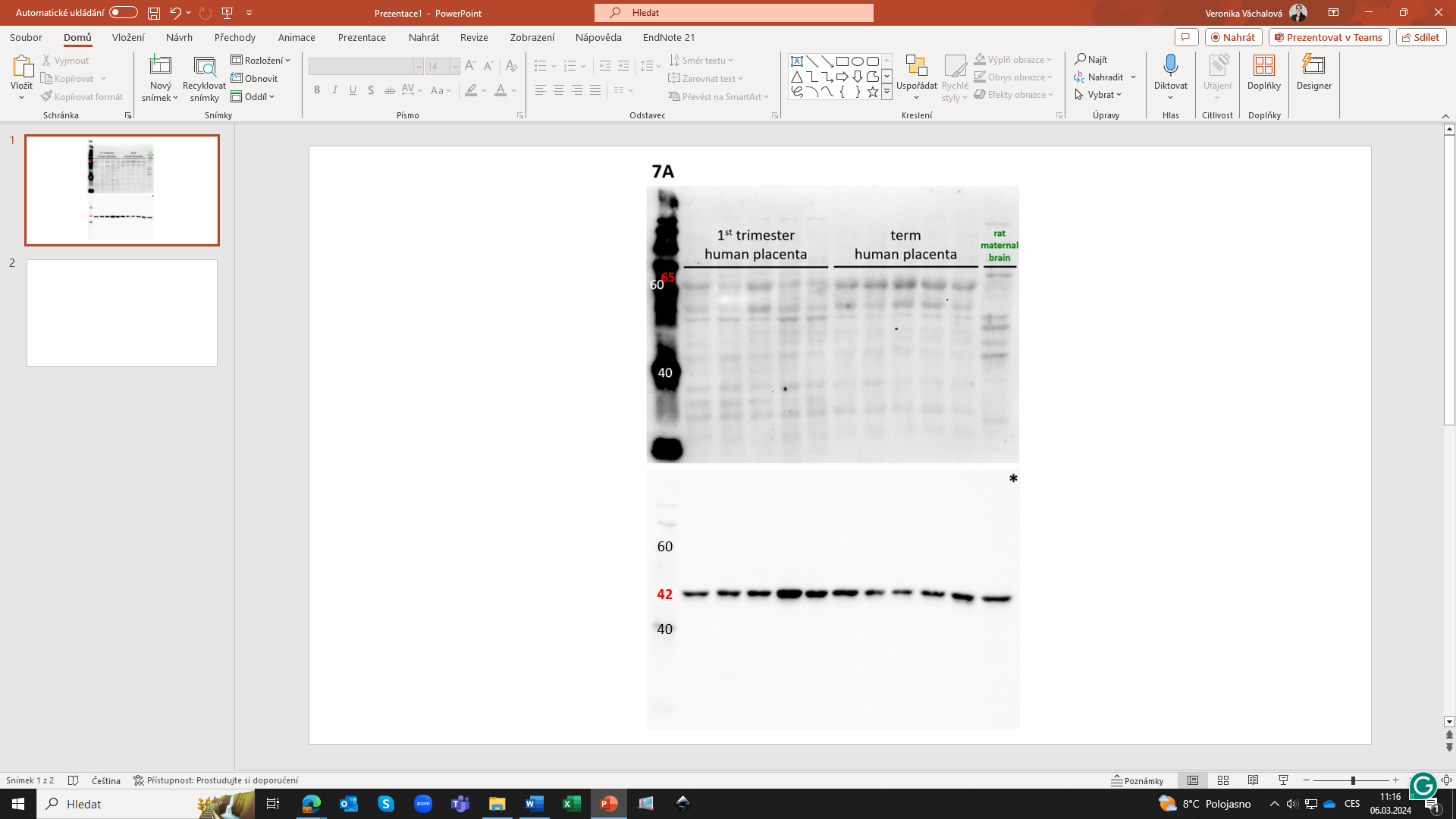

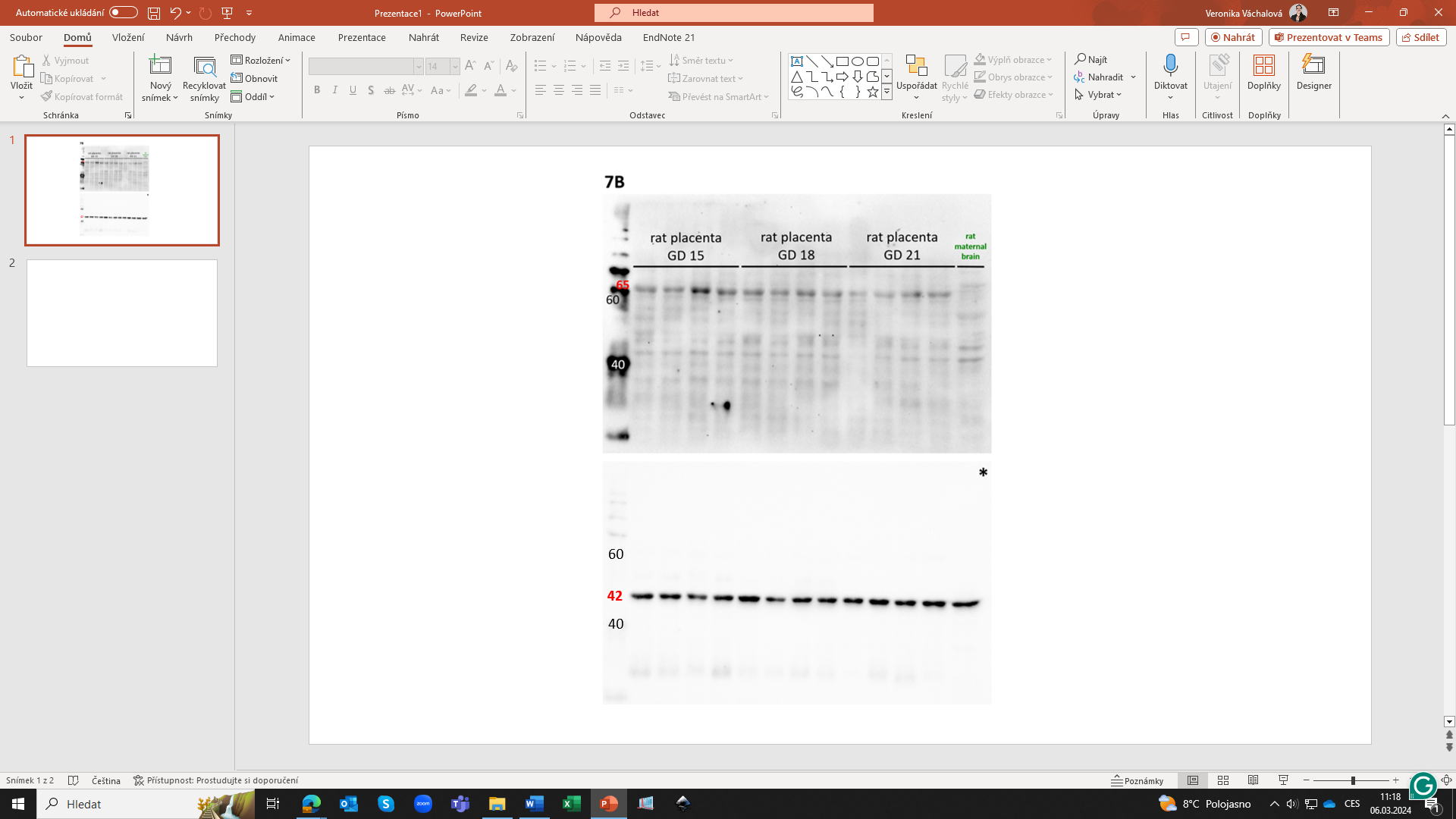


**Figure S3. Raw (uncropped) representative immunoblots from Western Blot analysis of expressed proteins.** Target proteins were analysed in human (PNMT – 5A, COMT – 6A, NET – 7A) and rat placentas (PNMT – 5B, COMT – 6B, NET – 7B). Rat adrenal gland, HepG2 cells, and rat maternal brain were used as positive controls and are indicated in green labelling for each target protein. Target protein size (kDa) is indicated in red label. Protein expression was normalized to β-actin as a loading control (shown as representative immunoblots labelled with asterisk *). No stripping of the initial antibody was performed before proceeding with the loading control.

**Figure S4. Comparison of gene expression profiles between term placenta samples from cesarean section and spontaneous birth deliveries.** Target gene expression was normalized against the geometric mean expression of GAPDH and B2M; n = 14 spontaneous/14 cesarean section. Data are shown as Tukey boxplots (1.5-times IQR); after log2 transformation.
